# Peptide-mediated delivery of donor mitochondria improves mitochondrial function and cell viability in human cybrid cells with the MELAS A3243G mutation

**DOI:** 10.1038/s41598-017-10870-5

**Published:** 2017-09-06

**Authors:** Jui-Chih Chang, Fredrik Hoel, Ko-Hung Liu, Yau-Huei Wei, Fu-Chou Cheng, Shou-Jen Kuo, Karl Johan Tronstad, Chin-San Liu

**Affiliations:** 1 Vascular and Genomic Center, Changhua Christian Hospital, Changhua, Taiwan; 2 Department of Biomedicine, University of Bergen, Bergen, Norway; 3 Department of Biochemistry and Molecular Biology, School of Life Sciences, National Yang-Ming University, Taipei, Taiwan; 4 Department of Medicine, Mackay Medical College, Taipei, Taiwan; 5 Stem Cell Center, Department of Medical Research, Taichung Veterans General Hospital, Changhua, Taiwan; 6 Department of Surgery, Changhua Christian Hospital, Changhua, Taiwan; 7 Department of Neurology, Changhua Christian Hospital, Changhua, Taiwan

## Abstract

The cell penetrating peptide, Pep-1, has been shown to facilitate cellular uptake of foreign mitochondria but further research is required to evaluate the use of Pep-1-mediated mitochondrial delivery (PMD) in treating mitochondrial defects. Presently, we sought to determine whether mitochondrial transplantation rescue mitochondrial function in a cybrid cell model of mitochondrial myopathy, encephalopathy, lactic acidosis, and stroke-like episodes (MELAS) disease. Following PMD, recipient cells had internalized donor mitochondria after 1 h, and expressed higher levels of normal mitochondrial DNA, particularly at the end of the treatment and 11 days later. After 4 days, mitochondrial respiratory function had recovered and biogenesis was evident in the Pep-1 and PMD groups, compared to the untreated MELAS group. However, only PMD was able to reverse the fusion-to-fission ratio of mitochondrial morphology, and mitochondria shaping proteins resembled the normal pattern seen in the control group. Cell survival following hydrogen peroxide-induced oxidative stress was also improved in the PMD group. Finally, we observed that PMD partially normalized cytokine expression, including that of interleukin (IL)-7, granulocyte macrophage–colony-stimulating factor (GM-CSF), and vascular endothelial growth factor (VEGF), in the MELAS cells. Presently, our data further confirm the protective effects of PMD as well in MELAS disease.

## Introduction

Mitochondria are organelles responsible for a large part of the cellular ATP production. These dynamic organelles have their own DNA, and are constantly adapting their function in accordance with the context-dependent needs of the cell^1^. Mitochondrial dysfunction is associated with many diseases, and typically leads to metabolic imbalance, cellular energy deficiency and ROS production^1^. Mitochondrial, myopathy, encephalopathy, lactic acidosis and stroke-like episodes syndrome (MELAS) is a genetic mitochondrial disease commonly caused by inherited point mutations in tRNA genes encoded by mitochondrial DNA (mtDNA). This results in defective synthesis of mitochondrial respiratory chain subunits and subsequent impairment of mitochondrial function^2^. The defects in mitochondrial function gives rise to a complex pathology that has severe consequences for patients. With the exception of mitochondrial replacement therapy, which only can be done on a newly fertilized oocyte, there is no curative treatment for MELAS or similar diseases. In the present study, we investigated if mitochondrial transplantation enabled by the cell-penetrating peptide Pep-1 rescue mitochondrial function in a cybrid MELAS model.

The mitochondria are double membrane organelles containing two enclosed compartments, the matrix (inner compartment) and the intermembrane space. The inner mitochondrial membrane is the site of the electron transport chain (ETC). Here electrons obtained from NADH and FADH_2_ are transported through four respiratory enzymes (CI-IV) via a series of redox reactions ending with the reduction of oxygen. This electron-transport drives the translocation protons from the matrix-side across the inner membrane, generating an electrochemical gradient (i.e. membrane potential). Reflux of protons through the ATP synthase complex (CV) releases energy used to phosphorylate ADP to ATP. Together, these processes are termed oxidative phosphorylation (OXPHOS)^1^. Mitochondrial bioenergetics are normally adapting to the physiological requirements of the cells, through regulation of oxidative pathways, mitochondrial biogenesis and mitochondrial dynamics^3^. Mitochondrial biogenesis serves to increase the oxidative capacity under conditions of insufficient ATP production^4^. Organelle fission and fusion processes are important in mitochondrial quality control, and involves fusion proteins such as OPA1^5^, MFN1 and MFN2^6^ and the fission proteins DRP1^7^ and Fis1^8^. Morphologic changes are seen in response to conditions of cellular stress. Mild energy deficiency, which may be due to increased ATP consumption in exercising skeletal muscle^9^ or sub-lethal inhibition of OXPHOS in cultured cells^10^ is associated with increased fusion and network complexity of filamentous mitochondria. Severe stress, which may be caused by pathology or toxin exposure, is associated with fragmented mitochondria, accompanied by aberrant ROS production and mitochondrial dysfunction^11, 12^. Specific degradation of dysfunctional mitochondria (mitophagy) has a crucial role in mitochondrial quality control, serving to sustain cellular energy homeostasis and prevent pathologic ROS production. Deficiencies in mitochondrial quality control are associated with neurodegenerative disorders such as Parkinson’s Disease^13^, and genetic mitochondrial diseases such as PolG mutations^14^ and in MELAS^15^.

Transfer of mitochondria between separate cells has been observed both *in vivo* and *in vitro*, a phenomenon that may support cell survival and protect against external stress^16–20^. The mechanisms involved in such intercellular organelle transfers are not fully understood, but nanoscale membrane containing tubes that form intercellular connections, tunneling nanotubes, have been reported to traffic mitochondria from mesenchymal stem cells and rescue damaged cells in culture^17, 19–21^. Another mechanism involving endocytosis and micro-vesicles containing mitochondria has been reported to transfer mitochondria between bone marrow derived mesenchymal cells and damaged alveolar epithelium *in vivo* ^19^. Such observations have given rise to studies addressing the possibility to rescue mitochondrial function by transplanting healthy mitochondria into cells with mitochondrial dysfunction. In an animal study, mitochondria injected directly into the heart after an induced ischemic insult protected against ischemia-reperfusion injury, reduced the infarction size and improved recovery^22^. In this study it was estimated that only 3–7% of the mitochondria were taken up by the cells, however, it was suggested that extracellular mitochondria also conferred some cardioprotection. Cell culture experiments have indicated that the ability to import exogenous mitochondria may vary between cell types. Whereas mtDNA depleted HeLa cells were found to take up exogenously added mitochondria^23^, the same was not seen mtDNA depleted A549 cells^16^. One study reported that mesenchymal stem cells were able to transfer mitochondria to mtDNA depleted cells, but not cybrid cells harboring pathogenic mtDNA with pathogenic mtDNA mutations, including the MELAS A3243G mutation^24^.

Cell-penetrating peptides (CPPs) have been employed to facilitate cellular uptake of substances such as nanoparticles, DNA and proteins^25, 26^. Such proteins have similar functions in nature, such as the HIV-1 protein, TAT, which is a CPP necessary for the entry of HIV-1 virus particles into human cells. Covalent coupling to TAT has been found to mediate import of functionally capable mitochondrial enzymes such as complex I subunits, which promoted recovery of vital cell functions^27^. Pep-1, another CPP, is able to translocate cargoes via non-covalent self-assembly mechanisms^28, 29^. Inside the cells, the cargo is released via an endocytosis-independent pathway^28, 29^. This ability of Pep-1 to act as a chaperone facilitating cellular uptake and intracellular release of cargo, may enable delivery of small organelles into the cell. Recently, we found that mitochondrial transplantation was facilitated when the isolated donor mitochondria were coated with Pep-1^30, 31^. Further, the studies showed that Pep-1 mediated mitochondrial delivery (PMD) improved mitochondrial functions in two cell models of the mitochondrial disease Myoclonic Epilepsy and Ragged Red Fibers (MERRF)^30, 31^.

Based on these findings, we here investigated if PMD is able to rescue mitochondrial properties in a MELAS cybrid cell model with MELAS A3243G mutation in mtDNA. Our aim was to clarify the effects of PMD on mitochondrial function and morphology to gain better understanding of the processes involved in restoring cell function. We also wanted to determine if Pep1-mediated mitochondrial transplantation influenced oxidative stress responses in the recipient cells because functional restoration could confer an increase of stress tolerance to the MELAS cybrid cells.

## Results

### Donor mitochondria are internalized into MELAS cybrid cells after PMD

**Figure 1:**
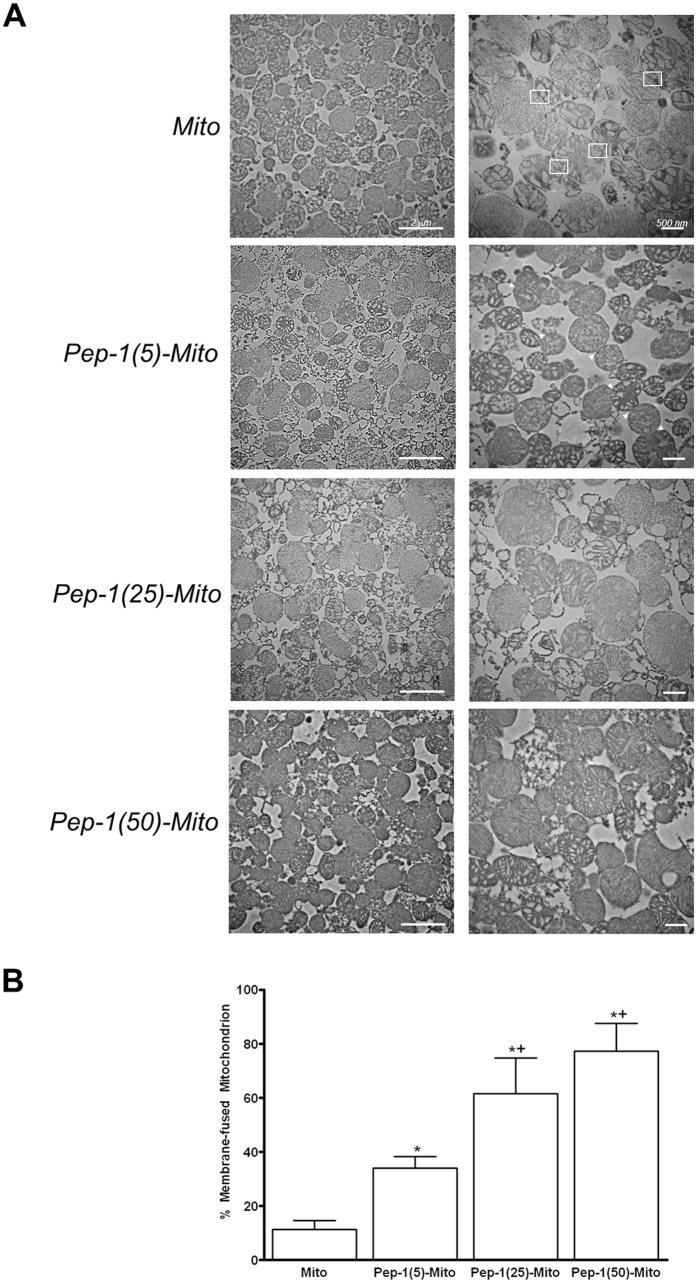
Transmission electron microscopy (TEM) analysis of mitochondria coated with various Pep-1 doses (5, 25 or 50 μM). (**A**) Mitochondria alone (Con); mitochondria coated with 5 μM Pep-1 (Pep-1(5)-Mito); mitochondria coated with 25 μM Pep-1 (Pep-1(25)-Mito); mitochondria coated with 50 μM Pep-1 (Pep-1(50)-Mito). Gap between outer membranes in the mitochondria alone group and contact between the outer membranes in the Pep-1(5)-Mito group is shown by block box and arrows, respectively. (**B**) Proportion of membrane-fused mitochondria in total mitochondria calculated from a group of images. *p < 0.05 compared to control group; ^+^ *p* < 0.05, compared to Pep-1(5)-Mito group.

To examine the direct effects of Pep-1 on the ultrastructure of isolated mitochondria, rat liver mitochondria were treated with Pep-1 (5, 25 or 50 µM), then analyzed by transmission electron microscopy (Fig. 1). A gap between the mitochondrial outer membranes was observed in mitochondria alone, as shown in Fig. 1A. The shape and membrane structure appeared unchanged in the presence of different concentrations of Pep-1, suggesting that Pep-1 does not affect mitochondrial integrity. However, close contact between the outer membranes was observed after Pep-1 labeling (indicated arrows in Fig. 1A). Membrane fusion was 34 ± 4.3%, 62 ± 13.3% and 77 ± 10.3 after treatment with 5, 25 and 50 µM Pep-1, relative to non-treated controls (11 ± 3.2) (Fig. 1B).

**Figure 2:**
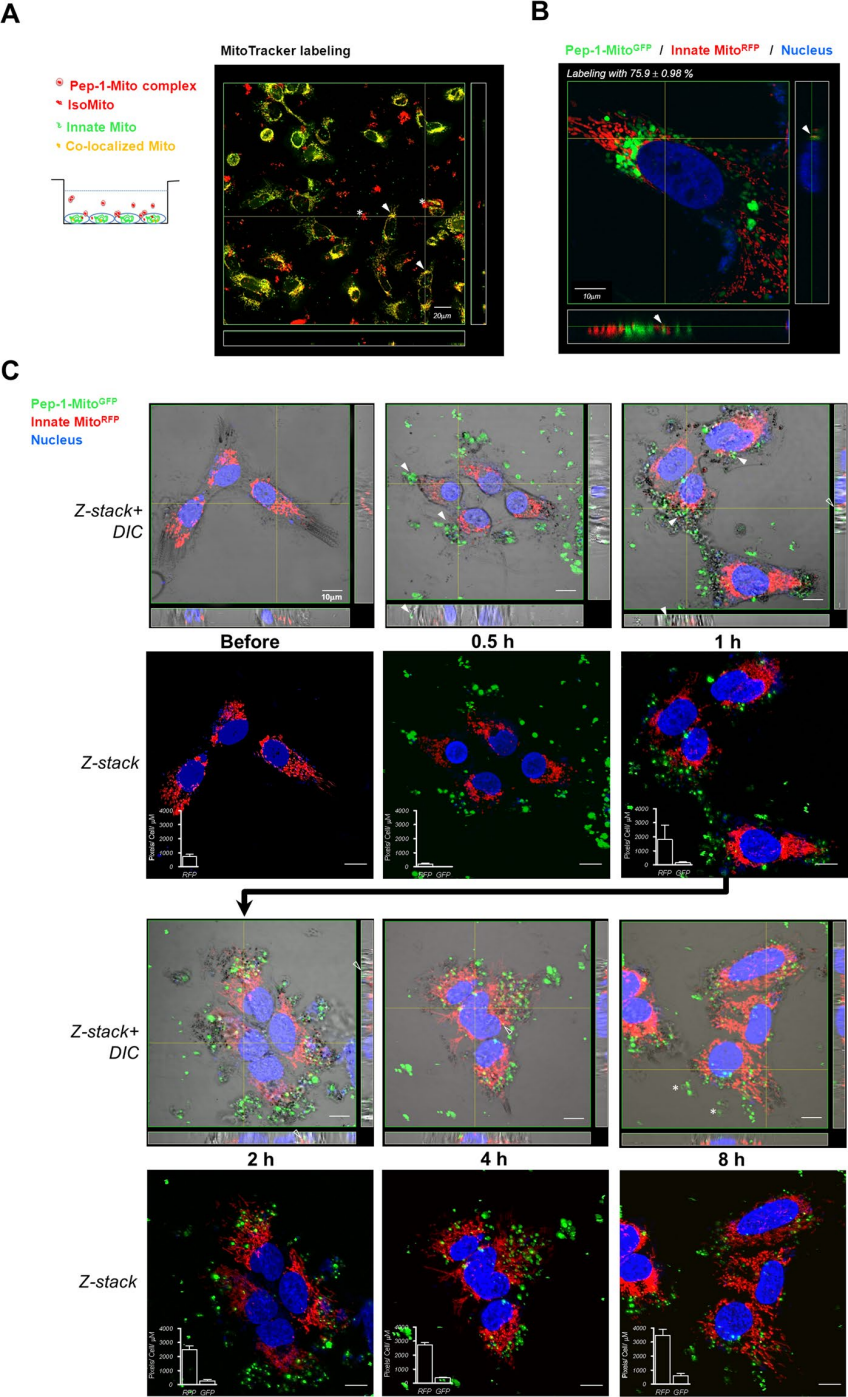
Translocation of Pep-1-labeled mitochondria (Pep-1-Mito) into MELAS cybrid cells. (**A**) Illustration showing mitochondrial translocation from medium into cells. Red (Mitotracker Red) and green (Mitotracker Green) fluorescence represent donor Pep-1-Mito and innate mitochondria, respectively. Asterisks indicate aggregates of Pep-1-Mito complexes suspended in medium. Reconstructed three-dimensional (3D) confocal images showing co-localization of donor and innate mitochondria inside MELAS cybrid cells, indicated by arrows (yellow fluorescence). (**B**) Mitochondria labeled with fluorescent proteins. Donor mitochondria tagged with GFP (Mito^GFP^, green fluorescence) and innate mitochondria tagged with HcRed1 (Mito^RFP^, red fluorescence). Nuclei were stained with DAPI (blue fluorescence). Donor Pep-1-labeled Mito^GFP^ (Pep-1-Mito^GFP^) inside recipient MELAS cybrid cells after 2 days; possible sites of fusion between donor and innate mitochondria (yellow fluorescence) are indicated by arrows. (**C**) 3D visualization of Pep-1-Mito^GFP^ internalization and innate Mito^RFP^ in MELAS cybrid cells tracked over time using confocal microscopy combined with differential interference contrast (DIC). Z-axis yields a longitudinal view showing Pep-1-Mito^GFP^ stuck to the cell membrane after 0.5 h and penetration into the cell after 1 h, as indicated by an arrow. Blank arrow indicates co-localization of Pep-1-Mito^GFP^ and Mito^RFP^ after 2 h and 4 h. Star symbol indicates decayed fluorescence of Pep-1-Mito^GFP^ suspended in medium after 8 h culture. Insert frame in z-stack image shows quantified Pep-1-Mito^GFP^ and Mito^RFP^ expression at each time point, represented by mean area of red fluorescence (pixel) per cell in same section thickness (μm).

To perform Pep-1-mediated mitochondrial delivery (PMD) on MELAS cybrid cells, donor mitochondria were isolated from 143B osteosacroma cybrid cells and labeled with MitoTracker Red immediately before delivery to enable organelle tracing. After an initial incubation of 4 h, red fluorescent mitochondria were observed both inside the MELAS cybrid cells and in mitochondrial aggregates in the medium, as shown using confocal microscopy and three-dimensional (3D) reconstructions (Fig. 2A). Within the cells, the red fluorescence overlapped significantly with green fluorescence from the mitochondria of the host cells (MELAS cybrid cells), which were labeled with MitoTracker Green prior to delivery. This co-localization of the two dyes could be due to fusion of host and recipient mitochondria, or to diffusion of the dyes between organelles. To address this, the experiment was repeated using GFP-tagged donor mitochondria (Mito^GFP^) and MELAS cybrid cells with HcRed1-tagged mitochondria. Confocal microscopy confirmed that donor mitochondria were internalized into the recipient cells, but did not appear to fuse with the host mitochondria during the course of the experiment; only a few examples of co-localized fluorescence could be identified (arrows in Fig. 2B).

Three-dimensional fluorescent imaging of live cells coupled with differential interference contrast (DIC) imaging was used to determine a time course for the entry of foreign mitochondria into host cells. Pep-1-labeled Mito^GFP^ (Pep-1-Mito^GFP^) mitochondria were attached to the cell membrane after an initial incubation of 0.5 h, but then had translocated into the cytoplasm after 1 h (Fig. 2C). More obvious co-localization of GFP and RFP was observed after 2 and 4 h of culture (blank arrows in Fig. 2C). After 8 h, extracellular Pep-1-Mito^GFP^ fluorescence suspended in the medium was significantly reduced (stars in Fig. 2C). Furthermore, the number of foreign Mito^GFP^ and innate Mito^RFP^, revealed by mean fluorescence area (pixels) per cell in same cell thickness, became enhanced with time (Fig. 2C, insert frame in z-stack image). Moreover, innate Mito^RFP^ in the rescued cells displayed a concomitant change in mitochondrial morphology, from a dot-like to a rod-like appearance.

**Figure 3:**
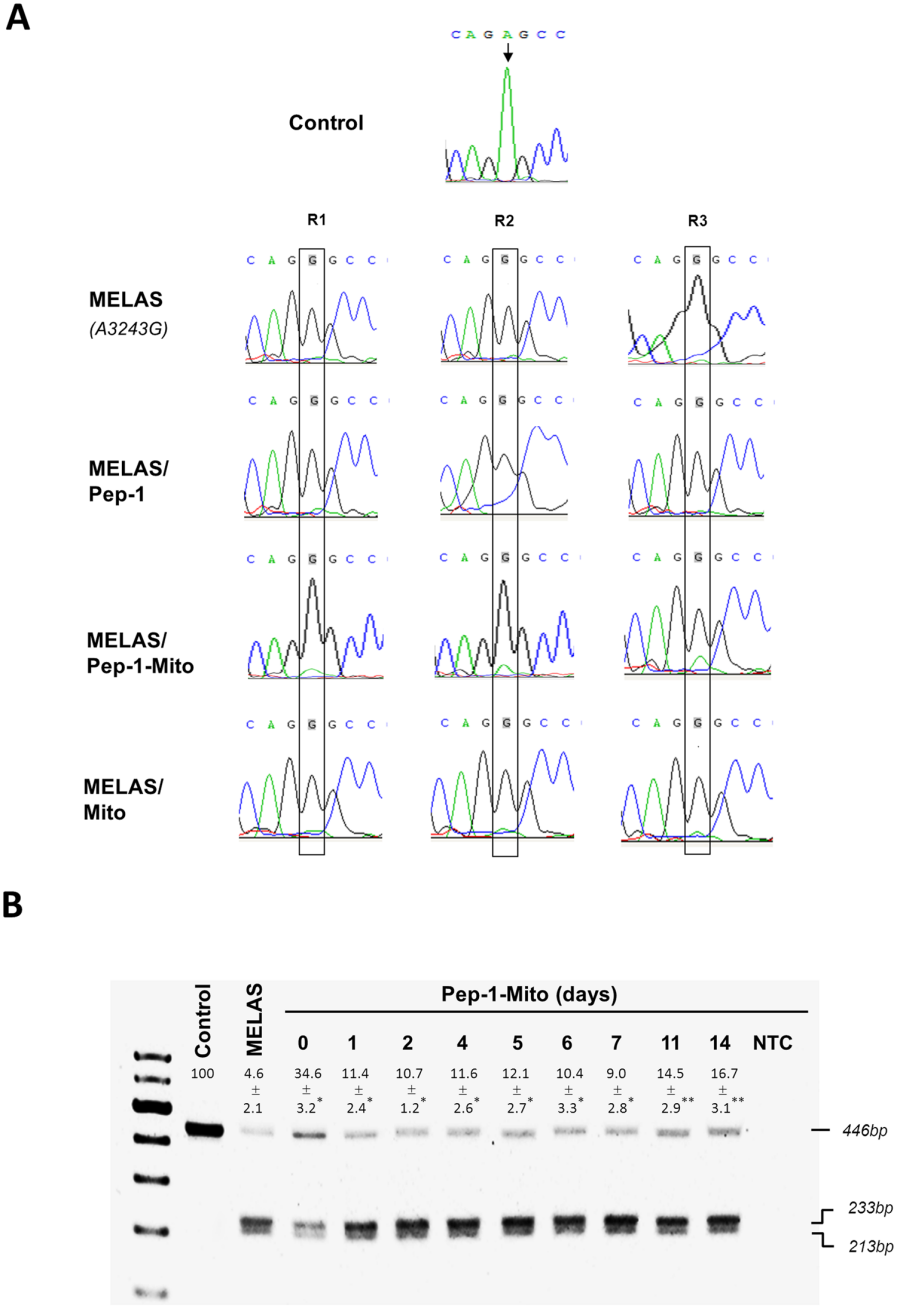
Sequence and genotyping analysis of Pep-1-Mito-treated MELAS cybrid cells. (**A**) Sequence electropherograms of mitochondrial MT-TL1 (tRNA^Leu^) showing an identical sequence change (A3243G) in each sample compared to control. Alterations in nucleotide sequence are indicated by arrows in each of three biological repeats (R). Presence of heteroplasmic mitochondrial DNA was found only in Pep-1-Mito samples. Control cybrid cells (Con); MELAS cybrid cells (MELAS); MELAS cybrid cells treated with Pep-1-coated mitochondria (Pep-1-Mito), carrier alone (Pep-1) or naked mitochondria (Mito). (**B**) Tracking mutated mtDNA in Pep-1-Mito-treated MELAS cybrid cells over time using restriction fragment length polymorphism (RFLP). RFLP from PCR-amplified fragments of mitochondrial tRNA^Leu^ indicating the mutation with two digested bands at 233 and 213 bp in MELAS cybrid cells, compared to a single 446 bp band in wild-type mtDNA-containing control cells. Percentage of wild-type mtDNA was shown as mean ± SD in control cells (Control), Untreated MELAS cybrid cells (MELAS) and following days of treatment; End of treatment with Pep-1-Mito complexes (Pep-1-Mito, day 0); Non template control (NTC). *p < 0.05, compared to MELAS group; **p < 0.05, compared to 7 days post treatment.

To confirm the presence of donor mitochondria inside the recipient MELAS cybrid cells, the mtDNA was sequenced. Donor wild-type mtDNA was detected in MELAS cybrid cells after PMD, as validated by DNA sequence analysis of the mitochondrial tRNA^Leu(UUR)^ gene at the A3243G mutation locus. This analysis was performed 48 h after incubation with Pep-1 carrier alone (Pep-1), naked mitochondria (Mito) or Pep-1-labeled mitochondria (Pep-1-Mito), and was compared to untreated MELAS cybrid cells. The presence of a heteroduplex mtDNA species (heteroplasmy) at position 3243 was found only in the Pep-1-Mito treated group (Fig. 3A). Moreover, PCR-RFLP analysis showed a significant increase in wild-type mtDNA content: 4.6 ± 2.1% in the untreated MELAS cybrid cells versus 34.6 ± 3.2% in the Pep-1-Mito treated cells at the end of the 48 h incubation period (day 0). Although wild-type mtDNA content was decreased in the subsequent stability period of 1–7 days, it was increased dramatically from approximately 9.0 ± 2.8% to 14.5 ± 2.9% after 11 days of treatment (Fig. 3B).

### Recovery of respiratory function in MELAS cybrid cells after PMD

**Figure 4:**
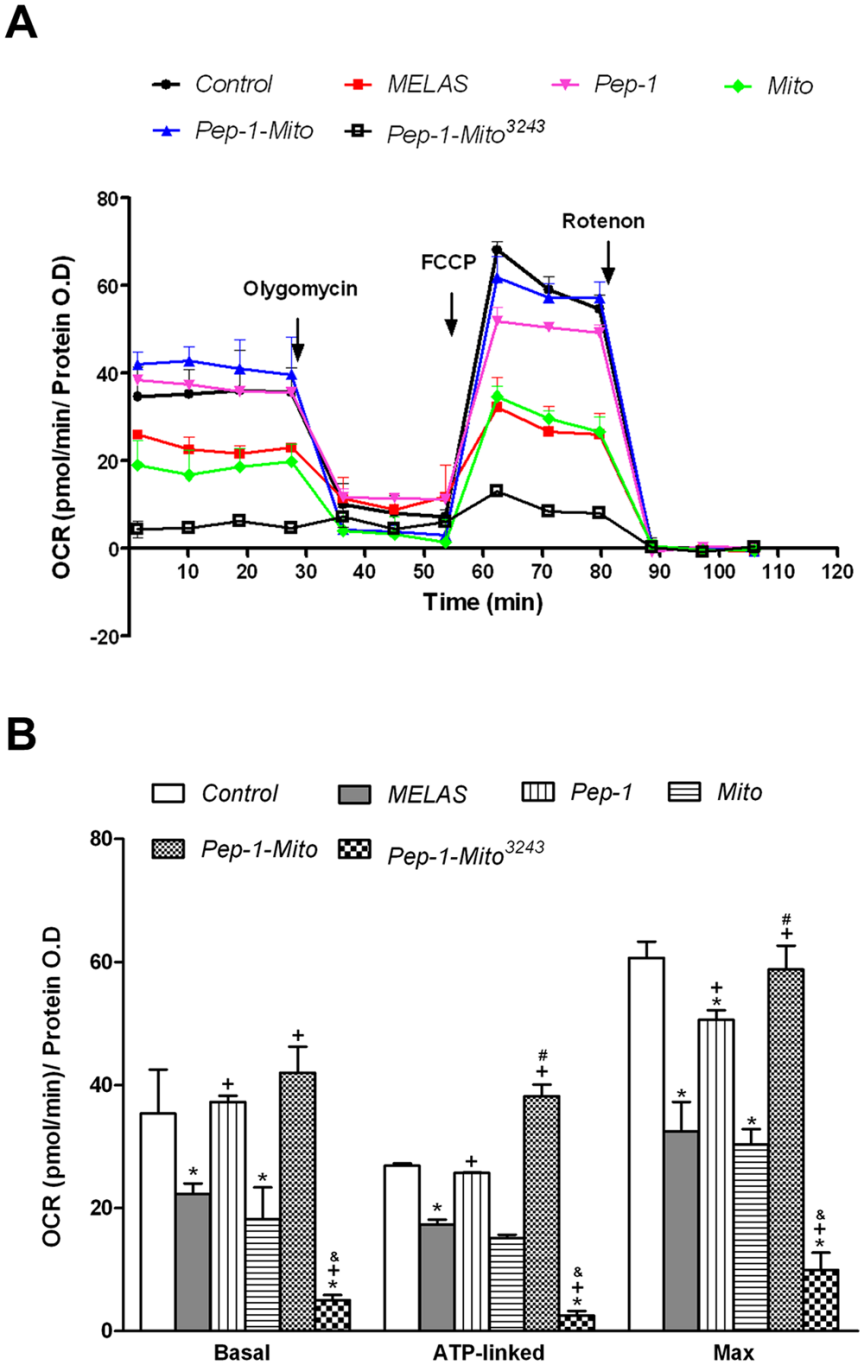
Seahorse X-24 analysis of oxygen consumption rate (OCR). (**A**) OCR measured after 4 days of treatment under normal conditions; after injection of oligomycin (1 μM); after injection of uncoupler FCCP (0.5 μM); after injection of electron transport inhibitor rotenone (2 μM) (n  =  5). (**B**) Basal respiration (Basal), ATP-linked and maximal respiratory capacity (Max) quantified by normalization of OCR to total protein OD value. All data were deducted from the non-respiration background for each group (OCR level post-rotenone treatment). Cell respiration measured in control cybrid cells (Con), MELAS cybrid cells (MELAS); MELAS cybrid cells treated with Pep-1 alone (Pep-1), naked mitochondria (Mito), Pep-1-labeled mitochondria (Pep-1-Mito) or Pep-1-labeled mitochondria carrying the mtDNA^3243^ mutation (Pep-1-Mito^3423^). *p < 0.05, compared to control group; ^+^ *p* < 0.05, compared to MELAS group; ^#^ *p* < 0.05, compared to Pep-1 group; ^&^ *p* < 0.05, compared to Pep-1-Mito group.

The effect of PMD on mitochondrial function after 4 days of treatment was investigated by monitoring oxygen consumption rate (OCR) in control cybrid cells, MELAS cybrid cells (MELAS), MELAS cybrid cells treated with Pep-1, Mito, Pep-1-Mito or Pep-1-labeled mitochondria with A3243G mutant mtDNA isolated from MELAS cybrid cells (Pep-1-Mito^3243^). OCR was measured in confluent cells after sequential treatment with specific inhibitors (oligomycin to inhibit ATP synthase, or the uncoupler carbonyl cyanide 4-(trifluoromethoxy)phenylhydrazone (FCCP), rotenone to inhibit complex I) (Fig. 4A). Basal OCR, ATP-linked OCR, and maximum capacity OCR were calculated by subtracting the non-respiratory background (post-rotenone rate) (Fig. 4B). Significantly lower rates of basal respiration, ATP-linked respiration, and maximal respiratory capacity were found in the untreated MELAS cybrid cells compared to control cells. PMD caused increases in basal respiration (1.7-fold increase), ATP-linked respiration (2.2-fold increase) and maximal respiratory capacity (1.8-fold increase), relative to the untreated MELAS group (Fig. 4B). It is contrary to MELAS cybrid cells treating with Pep-1-Mito^3243^ and its performance of respiratory function was even lower than the untreated. The result reflects the dependence of mitochondrial function on the manipulation of delivered mitochondria. No significant effects were in MELAS cybrid cells that received uncoated mitochondria. Treatment with Pep-1 alone also increased mitochondrial respiration, but the induction of ATP-linked and maximal respiration was significantly lower than after treatment with Pep-1-Mito.

### PMD alters expression of mitochondria shaping proteins and mitochondrial biogenesis in MELAS cybrid cells

**Figure 5:**
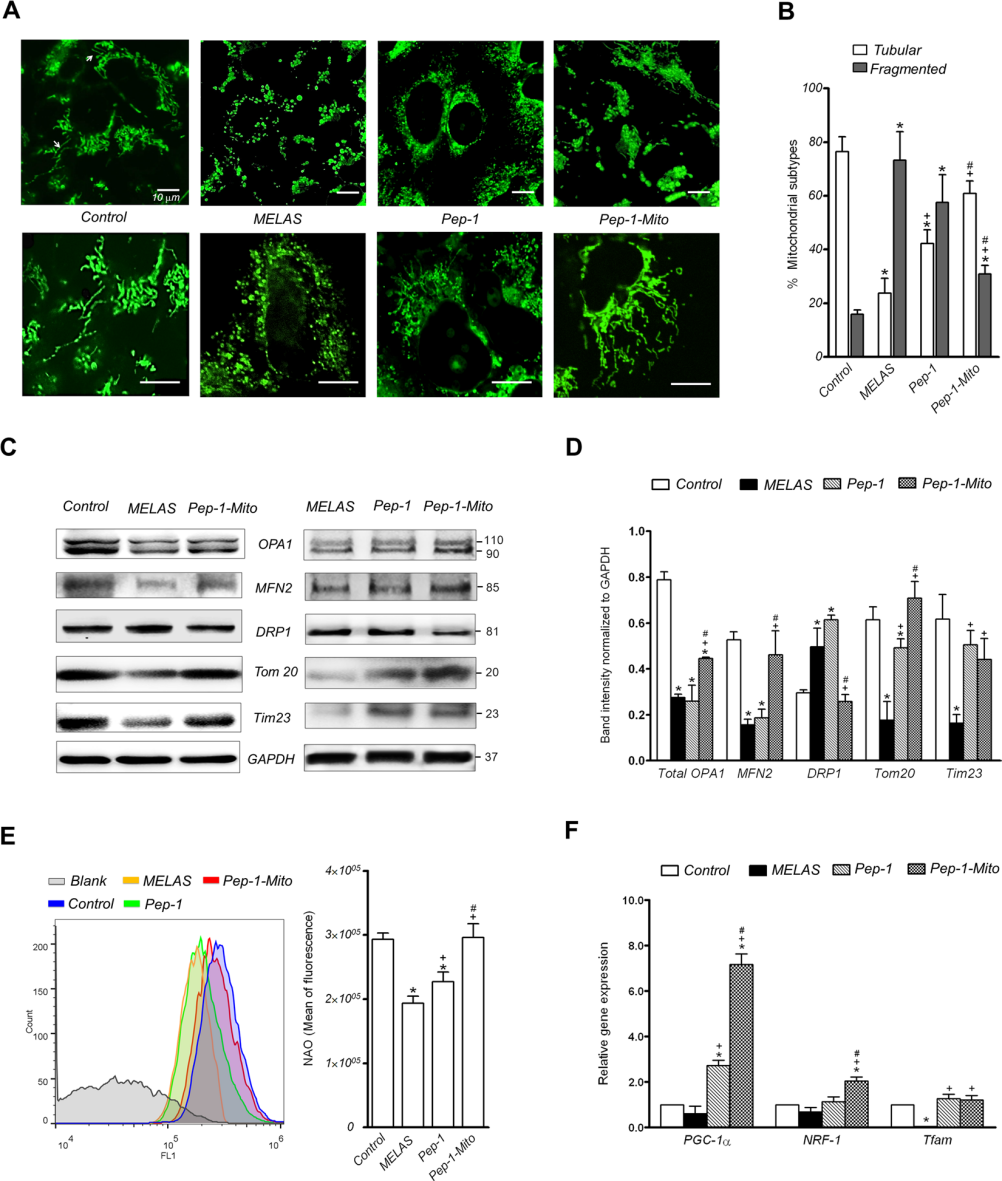
Mitochondrial morphology, mitochondria-shaping proteins and mitochondrial biogenesis in rescued cells after 4 days of treatment. (**A**) Morphology of mitochondria stained with MitoTracker Green observed by confocal microscopy. (**B**) Quantification of tubular and fragmented mitochondria was further analyzed using a semi-automatic system. Images from three independent areas containing approximately 200–300 mitochondria from about 6–8 cells each were analyzed from each group (n = 3). (**C**) Western blot analysis of mitochondrial fusion proteins OPA1 (including long and short OPA1 isoforms) and MFN2, fission protein DRP1 and mitochondrial marker proteins Tom20 and Tim23. (**D**) Protein expression was quantified by densitometry and normalized to GAPDH (n = 3). (**E**) Mitochondrial amount analyzed by NAO staining and flow cytometry. (**F**) Expression of mitochondrial biogenetic genes PGC-1α, NRF-1 and Tfam analyzed by RT-PCR. Relative expression levels were determined relative to β-actin. *p < 0.05, compared to control group; ^+^ *p* < 0.05, compared to MELAS group; ^#^ *p* < 0.05, compared to Pep-1 group; control cybrid cells (Con); MELAS cybrid cells (MELAS); MELAS cybrid cells treated with Pep-1 alone (Pep-1) or Pep-1-labeled mitochondria (Pep-1-Mito).

To examine the mitochondrial network in rescued MELAS cybrid cells, cells were treated with Pep-1-Mito and the morphology of mitochondria stained with MitoTracker Green was observed using confocal microscopy after 4 days, and compared with untreated cells (Fig. 5A and B). An abundant mitochondrial network and elongated morphology was observed in both the Pep-1 and Pep-1-Mito groups, compared with the MELAS group that exhibited dot- or sphere-like clusters, indicating mitochondrial fragmentation (Fig. 5A). However, Pep-1 treatment did not significantly reduce the fragmented proportion of mitochondria despite an increase in the proportion of tubular mitochondria from 24 ± 5.6% to 42 ± 5.1% (Fig. 5B). In contrast, PMD not only increased the proportion of tubular mitochondria from 24% to 61% but also decreased the proportion of spherical fragments from 73 ± 10.7% to 31 ± 3.2%. These mitochondria had a similar morphological pattern of tubular and fragmented mitochondria to those in the control group (Fig. 5B). To determine whether PMD affects the expression of mitochondrial-shaping proteins, we performed western blot analysis (Fig. 5C and D). Results revealed a consistent and significant decrease in mitochondrial fusion proteins OPA1 and MFN2, in MELAS cybrid cells compared to controls, and increased expression of the fission protein DRP1. These effects were associated with a significant reduction in expression of the mitochondrial marker proteins Tom20 and Tim23. These effects in MELAS cybrid cells were reversed after PMD, causing an increase in expression of OPA1, MFN2, Tom20, and Tim23, and a decrease in DRP1 expression, compared to untreated cells. Pep-1 treatment did not affect mitochondrial shaping proteins but did increase the expression of Tom20 and Tim23 protein in MELAS cybrid cells. Moreover, mitochondrial mass, revealed by NAO staining (Fig. 5E), and expression of mitochondrial biogenetic genes PGC-1α, NRF-1 and Tfam (Fig. 5F) were significantly restored after PMD, compared to untreated MELAS cybrid cells. Pep-1 treatment had the same effect, but the induction of mitochondrial biogenesis was not as good as with PMD treatment, particularly in terms of NRF-1 gene expression (Fig. 5E and F).

### PMD rescues MELAS cybrid cells from oxidative stress injury

**Figure 6:**
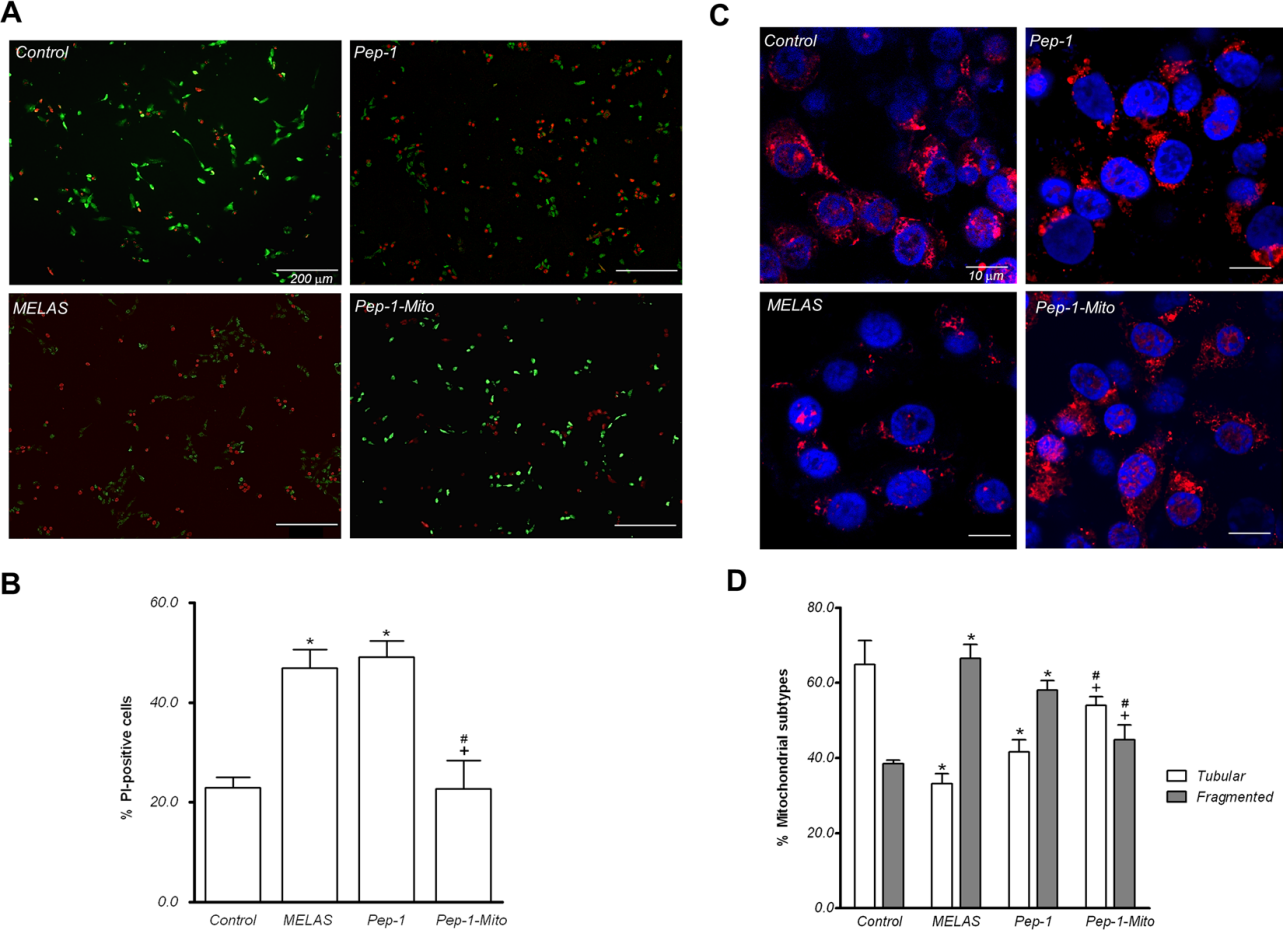
Cell viability and mitochondrial morphology after oxidation induced by hydrogen peroxide. (**A**) Double staining of live/dead cells with calcein-AM/PI was used to assess cell survival after 2 h treatment with 200 μM hydrogen peroxide. (**B**) Apoptotic cells quantified by counting PI-positive cells in images at the same magnification. (**C**) Morphology of mitochondria stained with MitoTracker Red observed by confocal microscopy of surviving cells under oxidative stress. (**D**) Quantification of tubular and fragmented mitochondria was further analyzed using a semi-automatic system. Images from three independent areas containing approximately 100–150 mitochondria from about 6–8 cells were analyzed from each group (n = 3). Control cybrid cells (Con); MELAS cybrid cells (MELAS); MELAS cybrid cells treated with Pep-1 alone (Pep-1) or Pep-1-labeled mitochondria (Pep-1-Mito). *p < 0.05, compared to control group; ^+^ *p* < 0.05, compared to MELAS group; ^#^ *p* < 0.05, compared to Pep-1 group.

To determine whether PMD improves oxidative stress tolerance in MELAS cybrid cells, cells were cultured in regular medium with 200 μM hydrogen peroxide. After 2 h, cell viability was assessed using Calcein-AM and PI staining. Results revealed a significantly higher number of dead cells (PI positive) in the MELAS cybrid cultures, compared to controls (Fig. 6A and B). PMD partially rescued this effect, but Pep-1 did not. Moreover, the morphology of mitochondrial network in cells that survived oxidative stress still showed an extensive tubular structure and fewer spherical fragments, in both control and Pep-1-Mito groups, compared to the MELAS group (Fig. 5C and D). There was no statistical difference in morphological changes between the Pep-1 and MELAS groups (Fig. 5D).

### Alterations in cellular cytokine production

To further evaluate the mechanisms of cellular stress after PMD, cytokine production was measured using a Bio-Plex Pro Human Cytokine 27-plex panel. Cytokines that showed a statistical difference between untreated MELAS cells and those that received Pep-1-coated or uncoated donor mitochondria, are shown in Table 1 (p < 0.05). PMD moderately influenced the cytokine profile in MELAS cybrid cells, by increasing the amounts of IL-1β, IL-8, IL-12 (p70), and VEGF, and reducing the amounts of IL-7 and GM-CSF, compared to untreated MELAS cybrid cells. These effects only partially reflect the cytokine profile in control cells. Treatment with Pep-1 protein alone had only minor effects on the cytokine profile. Delivery of uncoated mitochondria caused similar effects to PMD. These data suggest that PMD has moderate effects on the cytokine profile in MELAS cybrid cells, but this does not seem to be linked to the rescuing effects on mitochondrial function.

**Table 1:** Cytokine levels in cell lysates after 3 days of treatment.

Cytokines (pg/ml)	Control	MELAS	Pep-1-Mito	Pep-1	Mito
IL-1β	11.5 ± 0.35	15.1 ± 1.06*	22.6 ± 1.49^&^	17.6 ± 1.42	29.9 ± 2.89^&+^
IL-8	566.8 ± 18.46	558.6 ± 32.23	795.8 ± 74.34^&^	469.3 ± 14.84^&^	858.8 ± 0.42^&+^
IL-7	34.4 ± 2.63	60.4 ± 1.04*	54.7 ± 2.07^&^	63.9 ± 5.52	66.2 ± 4.80^+^
IL-12(p70)	110.0 ± 5.06	26.4 ± 4.19*	37.6 ± 5.85^&^	33.8 ± 14.76	46.9 ± 8.85^&+^
GM-CSF	666.3 ± 7.30	713.0 ± 18.67*	658.0 ± 13.24^&^	733.0 ± 28.21	675.9 ± 33.22
VEGF	657.8 ± 9.40	157.1 ± 16.50*	205.7 ± 7.02^&^	144.3 ± 41.34	187.8 ± 26.88

^*^ *p* < 0.05, compared to control group; ^&^ *p* < 0.05, compared to MELAS group; *p* < 0.05, compared to Pep-1-Mito group. Interleukin (IL)-1β, -7, -8, -12; Granulocyte-macrophage colony-stimulating factor (GM-CSF); Vascular endothelial growth factor (VEGF). All pooled data represent mean ± SD n = 3.

## Discussion

In the present study, PMD was shown to comprehensively improve mitochondrial function in a MELAS cybrid model to compare with the Pep-1 treatment which only useful aspect of mitochondrial respiration and biogenesis. Pep-1-Mito entered the MELAS cybrid cells 1 h after treatment, which resulted in increased numbers of both foreign and innate mitochondria within the cells. 2 days after treatment, clear expression of Pep-1-Mito^GFP^ could still be observed within the cells, and normal mtDNA was increased by approximately 6%, relative to untreated cells. A significant increase of normal mtDNA was observed immediately and 11-days post treatment in the cells. After 4 days of PMD treatment, mitochondrial function was restored, and was associated with enhanced mitochondrial mass, mitochondrial biogenesis and mitochondrial fusion versus fission. Recovery regulation depends on the function of the delivered mitochondria. Pep-1 treatment showed similar regulation to PMD, except for the mitochondrial dynamic balance. Only PMD treatment was able to effectively improve mitochondrial functionality, as shown by sustained cell survival and morphological elongation of the mitochondrial network in response to oxidative injury. The cytokine profile of the MELAS cybrid cells did not appear to be linked to the rescuing effects of PMD on mitochondrial function.

During normal growth conditions, consistent changes in mitochondrial morphology and mitochondrial shaping proteins OPA1, MFN2, and DRP1 were detected only after PMD. OPA1 and MFN2 are central to mitochondrial fusion, while DRP1 is a key player in mitochondrial fission. PMD not only dramatically increased mitochondrial elongation but also upregulated MFN2 and OPA1 expression, while DRP1 was significantly downregulated. These results suggest that a combination of increased mitochondrial fusion following a decrease in fission may help to improve resistance to oxidative stress-induced apoptosis^32, 33^. Indeed, increased cell survival against oxidative stress and a sustained mitochondrial tubular morphology was observed in cells following PMD, compared to untreated MELAS cells. OPA1-induced mitochondrial fusion is also associated with increased OXPHOS activity^34^. Furthermore, fusion of damaged and functional mitochondria results in the dilution of mutant mtDNA and compensation of mitochondrial components to preserve mitochondrial function. Our data also indicate that mitochondrial mass and biogenesis were consistently increased in MEALS cybrid cells after PMD, as shown by increased Tim23 and Tom20 expression and upregulation of mitochondrial biogenetic genes. Increased mitochondrial biogenesis and mitochondrial mass may support functional recovery of the cells, and could result from successful and efficient delivery of donor mitochondria and/or increased mitochondrial biogenesis. Thus, our data show that PMD improves cellular viability during oxidative stress, which is likely linked to its protective effects on mitochondrial dynamics and function. Moreover, we suggested that the improved mitochondrial quality could contribute to the beneficial effects of PMD, because the quality control process is intimately linked to the dynamic behavior of mitochondria, which undergo cycles of fusion and fission and communicate in a number of ways with their cellular environment^35^. An increase in respiration, number, and elongated morphology of mitochondria, without a dynamic balance between mitochondrial fusion and fission, was not enough to protect cells from oxidative stress-induced cell death, which was seen following Pep-1 treatment. Stimulating the quality control pathway to drive mitochondrial turnover has recently been considered as a therapeutic target for mitochondrial disorders^36^. We are beginning to explore the regulatory role of selective mitochondrial macroautophagy (mitophagy) in PMD therapy.

In MELAS, significant effects on respiration and ATP production have been shown to occur when the level of A3243G point mutation reaches above a threshold of 90% to 94%^37^. In our study, the presence of donor wild-type mtDNA in MELAS recipient cells was confirmed by genotyping. Since fluorescent mitochondria are due to fusion of GFP with the import sequence of the mitochondrial cytochrome c oxidase subunit 8 (COX8) rather than direct labeling with mtDNA, the amount of GFP signal does not accurately reflect the proportion of foreign mtDNA, especially after isolation from cells. A relatively high level of heteroplasmy (percentage of wild-type mtDNA) was observed immediately following PMD treatment (from 4.6 ± 4.6% to 34.6 ± 3.2%) and 11-days post treatment (from 9.0 ± 2.8% to 14.5 ± 2.9%) of stable culture. The proportion of normal mtDNA cannot be maintained at levels as high as at the start of treatment. We postulated that this could be related with mtDNA dilution with the deletion in the rapidly dividing cells^38^, or an increase in mtDNA replication within the cells^39^. The level of heteroplasmy has been reported to affect the transcription of genes involved glycolysis and OXPHOS-related genes in MELAS cybrid cells^40^ and determined the clinically relevant biochemical defect of diseases^41^. That could explain why mitochondrial respiration of MELAS cybrid cells was worse after delivery of Pep-1-Mito^3243^ in this study, due to a probable increase of the mutant proportion of mtDNA. Mitochondrial dysfunction in mitochondrial disease not only depends on the mtDNA heteroplasmy^37^ but also involved complex regulations such as nuclear gene mutations causing OXPHOS deficiency^41^. Thus, it is reasonable that the clinical expression threshold of mtDNA proportion can occur over a narrow range (generally a few percent) and various approaches have been used to induce a heteroplasmic shifting to below the mutation threshold^41^. Furthermore, the absolute amount of mtDNA in cybrid cells harboring both MELAS and wild-type mtDNA was found to correlate with increased respiration, even when the wild-type to mutant ratio remained low^42^. The increased content of non-mutated mtDNA in the MELAS cells seen after PMD in our study is consistent with the observed improvement in mitochondrial respiratory function. Furthermore, although mitochondrial function was not examined after long-term culture, we suggest that PMD could support the restoration of mitochondria in MELAS cells as well as in MERRF^31^, because normal mtDNA did not decline over time, but rather increased after 11 days of treatment.

Unlike other CPPs, such as Tat or penetratin, which are internalized through a form of endocytosis^43^, Pep-1 has a high affinity for lipidic membranes such that it can insert itself into the membrane bilayer and induce local destabilization to facilitate cell uptake^44^. Hence, it is not surprising that Pep-1 was able to alter the structure of the mitochondrial membrane in a dose-dependent manner. Pep-1 treatment not only induced fusion of two lipid bilayers of isolated mitochondria, but also increased tubular network formation in mitochondria within the cells, without affecting mitochondria-shaping proteins. To date, the effect of Pep-1 on mitochondrial function whether *in vitro* or *in vivo*, remains unclear. Recently, novel CPP targeting mitochondria (mtCPP) were developed to prevent mitochondrial damage by oxidative stress, which relied on their own antioxidant properties^45^. This study also demonstrated no cytotoxicity, even at high concentrations (100 µM)^45^. Thus, we suggest that membrane fusion caused by appropriate concentrations of Pep-1 rather improved function of mitochondria *in vitro* or *in vivo*. Moreover, we found that mitochondrial respiration as well as mitochondrial biogenesis and morphological elongation were increased in Pep-1-treated MELAS cybrid cells, although it failed to sustain cell survival after oxidative damage. We suggested that invalid regulation of mitochondrial dynamic in Pep-1 treatment could cause cells to lose the balance of mitochondrial fusion-fission to resist environmental stress via mitochondrial turnover^46^. Our previous study invalidated treatment with Pep-1 alone for neuroprotection in Parkinson’s disease^47^, which is in agreement with Meloni *et al*. who showed that the neuroprotective efficacy of CPPs is dependent on the diversity of CPPs and neuronal diseases^48^. Nonetheless, how dose Pep-1 regulate the genes and proteins involved in mitochondrial biogenesis and mitochondrial respiration are still worthy of further study.

Cellular stress due to mitochondrial function may affect immune responses, as observed in patients suffering from primary mitochondrial disorders^49^. We observed changes in the release of cytokines and chemokines, including IL-1β, -7,-12, GM-CSF and VEGF, from MELAS cybrid cells compared to control cybrid cells. Delivery of functional mitochondria by PMD partly attenuated the effects on IL-7, IL-12, GM-CSF and VEGF. However, it remains unclear whether mitochondrial transplantation has the potential to counteract some of the immunological aberrations in MELAS cells. There is also a potential for an inflammatory reaction against donor mitochondria *in vivo*. The presence of donor mitochondria caused increased levels of IL-1β, an important proinflammatory cytokine that is also involved in inflammatory disease^50^. Pep-1 alone did not induce significant changes in cytokine production in MELAS cybrid cells, and coating mitochondria with Pep-1 resulted in weaker upregulation of IL-1β, −8 and −12 compared to uncoated mitochondria. This could suggest that coating donor mitochondria with Pep-1 produces a weaker inflammatory reaction compared to uncoated donor mitochondria. A recent study using autologous mitochondrial transplantation in an animal model of ischemia-reperfusion injury found a decrease in inflammatory markers such as CRP, IL-6 and TNFα after transplantation compared to vehicle-only controls. In this study, CPP coating was not used to facilitate mitochondrial internalization, and only isolated mitochondria were injected into the target tissue^22^. Additionally, there was no development of anti-mitochondrial antibodies one month after transplantation, suggesting that at least autologous mitochondria are non-immunogenic *in vivo* ^22^.

In conclusion, our results suggest that PMD treatment can partly repair mitochondrial defects and improve cellular stress tolerance in cultured MELAS cells via regulation of mitochondrial quality, and are consistent with our previous studies using a MERRF cybrid model^30^. Future studies should be aimed at investigating the potential efficacy of PMD in animal models of mitochondrial disease.

## Materials and Methods

### Cell culture

Human 143B osteosacroma cybrid cells harboring wild-type (control cybrid) or A3243G mutant mtDNA (MELAS cybrid) were kindly donated by Prof. Y.H. Wei, as previously described^51^, with permission from the Institutional Review Board of National Yang-Ming University (approval No: IRB-1000030). All experimental procedures were approved by the Institutional Biosafety Committee of Changhua Christian Hospital (approval No: BS-T-004) and were carried out in accordance with the approved relevant guidelines and regulations. Cells were grown in Dulbecco’s modified Eagle’s medium (DMEM, high glucose) (Gibco/Invitrogen, Carlsbad, CA, USA) supplemented with 10% fetal bovine serum (FBS, GeneDireX, Las Vegas, NV, USA), 100 mg/mL sodium pyruvate (Gibco) and 1% PS (100 U/L penicillin G sodium, 100 mg/L streptomycin sulfate) (Gibco). For starvation experiments, cells were cultured in low glucose (1 g/L) DMEM (Gibco) supplemented with 2% FBS and 1% PS. Cells were cultured at 37 °C in a humidified incubator with 5% CO_2_.

### Mitochondrial labeling with fluorescent proteins

Donor (143B osteosarcoma cybrid) cells were transfected with a vector carrying a mitochondrial targeting fluorescent protein (GFP gene from *Aequorea coerulescens*, Ac-GFP). At 80% confluency, cells were transfected with 40 μg plasmid DNA encoding mitochondrial matrix-localized GFP (import targeting sequence of cytochrome c oxidase subunit 8, COX8) (Clontech, Palo Alto, CA, USA) using 60 μL PureFection transfection reagent (System Bioscience, SBI, Mountain View, CA, USA) according the manufacturer’s instructions. After 36 h, cells were transferred to normal growth medium for 4 h. Following G418 selection, cells containing GFP-tagged mitochondria (Mito^GFP^) were prepared for mitochondrial isolation. Mitochondria in MELAS cybrid cells were labeled with red fluorescence by transfecting with a plasmid encoding mitochondrial matrix-localized far-red fluorescent protein HcRed1 (Mito^RFP^) fused to the mitochondrial targeting sequence of COX8 (Clontech)^52^, as described above.

### Mitochondrial labeling with fluorescent dyes

Mitochondria were stained while within donor cells, prior to their isolation, using a MitoTracker dye (100 nM final concentration; MitoTracker Red CMXRos or MitoTracker Green FM; Invitrogen-Molecular Probes, Eugene, OR, USA) for 20 min in a 37 °C incubator. Cells were then washed twice with PBS to remove any remaining dye.

### Mitochondrial isolation and Pep-1-mediated delivery

Purification of mitochondria, Pep-1 conjugation and isolation of mitochondria were conducted as previously described^30, 31^. Donor mitochondria were isolated from human 143B osteosacroma cybrid cells harboring either wild-type or A3243G mutant mtDNA, according to experimental needs. The isolation procedure was performed using a mitochondria isolation kit for cultured cells, according to the manufacturer’s protocol (Thermo Fisher Scientific, Carlsbad, CA, USA). Briefly, donor cells were detached from the plate by trypsinization, harvested, and centrifuged at 1000 rpm for 5 min. Twenty million cells were collected for each mitochondrial isolation procedure. Cells were washed twice in ice-cold PBS before beginning the isolation procedure. Cells were homogenized on ice in isolation reagent supplemented with proteinase inhibitor (EMD Millipore, Billerica, MA, USA) using a glass Dounce tissue grinder. After adding an equal volume of separation buffer, the homogenate was centrifuged at 3000 rpm for 10 min at 4 °C to separate cytosol and intact cells. To exclude other organelles, the cytosol was centrifuged at 3000 × g for 15 min, the pellet was suspended in separation buffer and collected by centrifugation at 12000 × g for 3 min, at 4 °C. Protein concentration was determined using a bicinchoninic acid (BCA) assay kit (Pierce Biotechnology, Rockford, IL, USA). For delivery of mitochondria, 200,000 cells were plated onto 10-cm dishes and received 105 μg mitochondria coated with Pep-1 (Anaspec, San Jose, CA, USA) (Pep-1-Mito) at the appropriate weight ratio (Mito/Pep-1, 1.75:1). Pep-1 concentration was about 50 μM. After 2 days of exposure to Pep-1-Mito, recipient cells were washed twice with PBS before further culturing in regular medium.

For mitochondrial isolation from mouse liver, mice was sacrificed and perfused with 50 mL 0.9% NaCl. Livers were sliced and homogenized using a Dounce homogenizer with 5 mL isolation buffer (0.25 M sucrose, 0.5 mM EGTA, 3 mM HEPES-NaOH, pH 7.2) with protease inhibitors. All operations were carried out at 0–4 °C. The homogenate was centrifuged for 15 min at 1000 × g. The supernatant was collected, layered onto a sucrose gradient and centrifuged for 30 min at 35000 × g. The sucrose concentration gradient was from 30%, 40% and 55% for isopycnic banding and from 40% to 55% when mitochondria were collected in a pellet. The pellet was resuspended in isolation buffer and centrifuged twice at 13000 × g for 3 min each. Resuspended rat mitochondria were prepared for ultrastructural analysis after Pep-1 conjugation as described below.

### Transmission electron microscopy

Mitochondria isolated from mouse liver (5 mg/mL) were conjugated with 5 (Mito/Pep-1, 339:1), 25 (Mito/Pep-1, 68:1) or 50 μM (Mito/Pep-1, 34:1) Pep-1 to observe mitochondrial ultrastructure. Mixtures were centrifuged at 9000 × g for 2 min. Pellets were then fixed in 2.5% glutaraldehyde in 0.1 M phosphate buffer (pH 7.2) for 4 h at room temperature as around 20 to 25 °C. After three rinses in 0.1 M phosphate buffer for 15 min each, samples were dehydrated through a graded ethanol series (30%, 50%, 70%, 85%, 90% and 100%) for 20 min each. Samples were infiltrated with LR white resin in a gelatin capsule and stored at 4 °C for 48 h. Capsules were polymerized at 60 °C for 16 h, before cutting 70 nm ultrathin sections (Leica EM UC7, Germany). Sections were viewed using a transmission electron microscope (TEM) (Hitachi H-7000, Japan). The proportion of membrane-fused mitochondria was calculated by counting the number of mitochondria with any one side in contact with another within the total number of mitochondria in each image. Approximately 200 mitochondria were counted in an individual image.

### Confocal microscopy

For visualization of mitochondria, cells were seeded onto a chamber slide after 4 days of treatment (µ-Slide 8 well, Ibidi GmbH, Martinsried, Germany), stained with Mitotracker Green (250 nM) (Invitrogen) and placed in an incubator at 37 °C for 40 min. After staining, the remaining dye was removed, and stained cells were mounted onto a perfusion chamber in culture media and imaged at 37 °C using an Olympus FluoView FV 1200 confocal microscope (Olympus, Tokyo, Japan).

To track the internalization of Pep-1-labeled Mito^GFP^ (Pep-1-Mito^GFP^) in MELAS cybrid cells expressing Mito^RFP^, three dimensional (3D) reconstructions were generated from confocal microscopy images combined with difference interference contrast (DIC). To determine the distribution of Pep-1-Mito^GFP^ and innate Mito^RFP^ within host cells, line scans through the z-axis were integrated to yield a longitudinal view, which were combined (0.55 μm z-steps) using Olympus Fluoview Viewer software. Innate Mito^RFP^ and internalized Pep-1-Mito^GFP^ in host cells were quantified at different time points by calculating the mean area of red and green fluorescence (pixels) per cell within the same section thickness (μm) using ImageJ Software. The expression of Mito^RFP^ and Pep-1-Mito^GFP^ in the incomplete cells was excluded to calculate in the z-stack images.

### Genotyping

DNA was extracted from cultured cells using a Qiagen DNeasy kit (Qiagen, Valencia, CA, USA). Presence of the MELAS A3243G mutation in mtDNA was determined using a polymerase chain reaction (PCR)-restriction fragment length polymorphism (RFLP) analysis with Apa I, as previously described^53^. A DNA fragment of 446 bp encompassing a putative mutation at nucleotide position (np) 3243 was amplified by PCR using a forward primer from np 3010 to 3029 and a reverse primer from np 3436 to 3456. Amplification was performed for 36 cycles of denaturation at 94 °C for 1 min, annealing at 54 °C for 1.5 min, and elongation at 72 °C for 1 min. DNA was then digested at 25 °C overnight in 3 μL 10 × BSA, 3 μL NEB Buffer 4 and 0.8 μL Apa I (New England Biolabs, Ipswich, UK). The mtDNA carrying the A3243G mutation was cut by Apa I resulting in digestion bands of 233 and 213 bp. In the absence of the mtDNA mutation, the PCR product would not be cut and would remain at its original size of 446 bp. Digestion of the mutant DNA did not affect wild-type DNA. Digested products were visualized by electrophoresis on a 4% agarose gel with 0.3 μg/mL ethidium bromide. The proportion of mtDNA with the A3243G mutation in each group was calculated by normalizing the mutant DNA fragments of length 233 and 213 bp to total band intensity using GelPro Analyzer (Media Cybernetics, Silver Spring, MD, USA).

The mtDNA A3243G mutation ratio was determined by sequencing the PCR products, which were randomly sampled then sequenced using an ABI 3130xl genetic analyzer and a BigDye Terminator v1.1 cycle sequencing kit (Applied Biosystems, Foster City, CA). To confirm the sequencing results, the position 3243 A to G mutation of mtDNA heteroplasmy was performed by using an automated DNA sequencer (ABI Prism 310 Genetic Analyzer, PE Applied Biosystems, Foster City, CA, USA) according to the manufacturer’s instructions.

### Mitochondrial respiration analysis

Mitochondrial respiration was measured using a Seahorse Xfe24 Analyzer (Agilent Technologies). After 4 days of treatment, cells were seeded onto a XF24 microplate (30,000 cells/well) in normal culture medium and cultured for 16–18 h. The culture medium was then replaced by analysis medium (DMEM, high glucose, without FBS, and sodium bicarbonate) and incubated without CO_2_ for 1 h before analysis according to the manufacturer’s protocol. Basal oxygen consumption rate (OCR) was measured first, then after sequential injections of three compounds that affect bioenergetics: 1 μM oligomycin (Sigma, St. Louis, MO, USA), 0.5 μM carbonyl cyanide 4-(trifluoromethoxy)phenylhydrazone (FCCP) (Sigma) and 2 μM rotenone (Sigma). After completion of the analysis, protein was measured using a BCA assay (Pierce Biotechnology). Results were normalized to the protein OD value of corresponding wells.

### Analysis of mitochondrial morphology

For visualization of mitochondria, stained cells were mounted onto a perfusion chamber in culture media and imaged at 37 °C using an Olympus FluoView FV 1200 confocal microscope (Olympus, Tokyo, Japan). Subtyping of mitochondrial morphology was quantified using an automatic classification system according to Peng *et al*.^54^. After semi-automatic segmentation of cell micrographs, mitochondria were classified into six distinct subtypes (small globe, swollen globe, straight tubule, twisting tubule, branch tubule and loop) using automatic classification software. Considering the error of automatic analysis caused by fuzzy staining of mitochondria due to excessive dye loading, the mitochondrial loop subtype was excluded. The proportion of tubular mitochondria was calculated by totaling the straight tubule, twisting tubule and branch tubule mitochondrial populations. The proportion of fragmented tubular mitochondria was calculated by totaling the small globe and swollen globe mitochondrial populations. Micrographs from three independent areas per group were analyzed, and about 200–300 mitochondria from about 6–8 cells in each image was calculated.

### Western blot

Following incubation, treated cells were washed with PBS and collected into RIPA buffer (50 mM Tris-Cl pH 7.4, 150 mM NaCl, 1% NP40, 0.25% Na-deoxycholate, 1 mM PMSF) (Pierce Biotechnology) containing protein inhibitor (Sigma) and phosphatase inhibitor cocktails (Pierce Biotechnology). Cells were incubated on ice for 20 min then homogenized. Extracts were spun down at 14,000 × g for 10 min at 4 °C and supernatants analyzed using the Bradford assay. Total cell lysates were separated by SDS–PAGE, and transferred onto Immobilon-P membranes (Millipore, Bedford, MA, USA). Membranes were incubated with primary antibodies: anti-OPA1 (NOVUS Biologicals, Littleton, CO, USA; 1:1000), anti-MFN2 (NOVUS Biologicals, 1:1000), anti-DRP1 (Santa Cruz, CA, USA; 1:500), anti-Tom 20 (Santa Cruz; 1:1000), anti-Tim 23 (Santa Cruz; 1:1000) and anti-GAPDH (Santa Cruz; 1:1000), followed by incubation with horseradish peroxidase (HRP)-conjugated secondary antibodies (goat anti-mouse-HRP or goat anti-rabbit; Jackson ImmunoResearch, PA, USA; 1:50000). Signal was detected using an ECL western blotting detection system (Millipore).

### Mitochondrial mass assay

Mitochondrial mass was measured using a nonyl acridine orange (NAO) stain. Treated cells were incubated with medium containing 100 nM NAO for 20 min at 37 °C. Cells were washed twice in PBS, centrifuged at 1200 rpm for 5 min, and fluorescence was detected using flow cytometry (Beckman Coulter, CA, USA). Data were analyzed using Flowjo software (TreeStar, OR, USA).

### RNA extraction and quantitative real-time RT-PCR analysis

Total RNA was purified from cells using a NucleoSpin RNA kit (Macherey-Nagel, Düren, Germany) and reverse-transcribed using a Transcriptor First Strand cDNA Synthesis kit (Roche Applied Science, Indianapolis, IN, USA). Expression of mitochondrial biogenetic genes including peroxisomal proliferator activator receptor gamma coactivator 1a (PGC-1α, forward primer: 5′ GGAGAGGCAGAGGCAGAAGG-3′ and reverse primer: 5′-AAGCATCACAGGTATAACGGTAGG-3′), nuclear respiratory factor 1(NRF-1, 5′-CCGTGGCTGATGGAGAGGTGGAAC-3′ and forward primer: 5′-CTGATGCTTGCGTCGTCTGGATGG-3′) and mitochondrial transcriptional factor A (Tfam, forward primer: 5′-GGAGTTGTGTATTGCCAGGAG-3′ and reverse primer: 5′-CTTCGGAGAAACGCCATCG-3′) were determined by quantitative real-time RT–PCR using SYBR Green PCR Master Mix (Roche Applied Science) and an ABI Prism 7300 system (Applied Biosystems). Reaction parameters were 2 min hold at 50 °C, 10 min hold at 95 °C, followed by 40 cycles of 15 s at 95 °C, and 1 min at 60 °C. All measurements were performed in triplicate. mRNA expression was normalized to β-actin, and presented as the relative expression level.

### Cell viability after oxidative stress

Cells were seeded onto 24-well plates with 2000 cells per well, allowed to adhere overnight, then treated with 200 µM hydrogen peroxide for 2 h in regular medium at 37 °C. To examine cell viability after oxidative induction, cells were washed twice with PBS and double stained with 1 μM Calcein-AM (Calcein acetoxymethyl ester) (Invitrogen, South San Francisco, CA, USA) and 1 μM propidium iodide (PI) (Invitrogen) at 37 °C for 30 min. Viable and apoptotic cells were observed using an inverted fluorescence microscope (IX81, Olympus, Tokyo, Japan).

### Multiplex cytokine assay

Three days post-treatment, cell lysates were extracted using RIPA buffer (Millipore) containing proteinase inhibitor (Millipore) at 4 °C. Protein concentration was measured using a BCA assay kit (Pierce Biotechnology). Multiplex cytokines in cell lysates with a final protein concentration of approximately 2 mg/mL were analyzed using a BioplexTM Pro-human cytokine 27-plex panel (Bio-Rad Laboratory, Hercules, CA, USA), according to manufacturer’s guidelines. Briefly, 50 μL antibody-conjugated beads were added to a 96-well filter plate and adhered using vacuum filtration. Fifty microliters of pre-diluted standards, blanks or samples were individually transferred into the wells after washing. The plate was incubated at room temperature on a shaker at 850 rpm for 30 min. After washing, 50 μL pre-diluted streptavidin-conjugated PE was added and incubated at room temperature on a shaker for 10 min. After washing, 125 μL assay buffer was added to each well, and the plate was placed onto a shaker for 30 seconds. The concentration of each cytokine was determined using a BioRad BioPlex 200 instrument with BioManager v6.0 software (Bio-Rad). All groups were run with triplicate samples.

### Statistical analysis

Comparison of two experimental conditions was evaluated using the paired Student’s t-test. A difference of *p* < 0.05 was considered statistically significant. All experiments were repeated at least three times with triplicate samples. Data are presented as mean ± SD.
